# Recent Progress on Therapeutic Vaccines for Breast Cancer

**DOI:** 10.3389/fonc.2022.905832

**Published:** 2022-06-06

**Authors:** Lianru Zhang, Xipeng Zhou, Huizi Sha, Li Xie, Baorui Liu

**Affiliations:** ^1^The Comprehensive Cancer Centre of Drum Tower Hospital, Medical School of Nanjing University & Clinical Cancer Institute of Nanjing University, Nanjing, China; ^2^Department of oncology, Yizheng People’s Hospital, Yangzhou, China

**Keywords:** breast cancer, cancer vaccines, cancer immunotherapy, clinical trials, concurrent therapies

## Abstract

Breast cancer remains the most frequently diagnosed malignancy worldwide. Advanced breast cancer is still an incurable disease mainly because of its heterogeneity and limited immunogenicity. The great success of cancer immunotherapy is paving the way for a new era in cancer treatment, and therapeutic cancer vaccination is an area of interest. Vaccine targets include tumor-associated antigens and tumor-specific antigens. Immune responses differ in different vaccine delivery platforms. Next-generation sequencing technologies and computational analysis have recently made personalized vaccination possible. However, only a few cases benefiting from neoantigen-based treatment have been reported in breast cancer, and more attention has been given to overexpressed antigen-based treatment, especially human epidermal growth factor 2-derived peptide vaccines. Here, we discuss recent advancements in therapeutic vaccines for breast cancer and highlight near-term opportunities for moving forward.

## Introduction

Breast cancer (BC) is the leading cause of cancer worldwide (1). Although there has been an increase in the overall survival rate in BC because of improvements in early-stage diagnosis and targeted therapies, almost all metastatic tumors develop drug resistance and cannot be cured. It is still a difficult problem to reduce the recurrence rate of early breast cancer and to prolong the survival time of advanced breast cancer. Immune-based interventions could be a beacon of hope to decrease morbidity and mortality of cancer. Although immune checkpoint inhibitors (ICIs) have been proven to increase the survival rate in lung cancer, melanoma, gastric cancer and so on, the indications of ICIs for the treatment of BC are only focused on first-line and neoadjuvant therapy for triple-negative breast cancer (TNBC) (2) to date.

The tumor microenvironment (TME) plays a crucial role in the recognition and prevention of cancer and early eradication. The TME may also interact with tumor cells and promote the progression of cancer. The immunoediting hypothesis describes the dynamic interaction between the immune system and tumor cells in three phases: elimination phase, equilibrium phase and escape phase (3). Tumor cells that avoid immune recognition and elimination steps enter the escape phase and present a clinically detectable tumor. The advantage of active immunotherapy is to develop a protective effect against tumor tissue, modifying the immune microenvironment and resetting the immune system to an antitumor surveillance status. Therapeutic cancer vaccines led by neoantigens are hotspots of active immunotherapy. Combination strategies with ICIs have shown clinical benefits in multiple types of cancer (4, 5). To date, only one vaccine named sipuleucel-T has been approved by the FDA and is used to treat metastatic castration-resistant prostate cancer in a limited group of nearly asymptomatic patients (6). No BC vaccine has been approved for clinical use. BC is a heterogeneous disease and can be classified into 4 common groups: luminal A, luminal B, human epidermal growth factor 2 (HER2)-positive, and TNBC (7). BC is traditionally considered a poorly immunogenic tumor. However, recently published data on TNBC have shown that a significant number of tumor infiltrating lymphocytes infiltrate TNBC tissues (8), indicating that an immunotherapeutic approach may be suitable for this hard-to-treat malignancy. A series of clinical trials for TNBC vaccines are underway. In addition, increasing numbers of clinical trials are being conducted demonstrating that vaccination is capable of inducing an antitumor-specific response in BC. In this review, we discuss recent progress on therapeutic vaccines from the perspective of tumor development and clinical data, and a blueprint for personalized vaccines is also presented.

## Spectrum of Vaccine Targets

**Figure 1:**
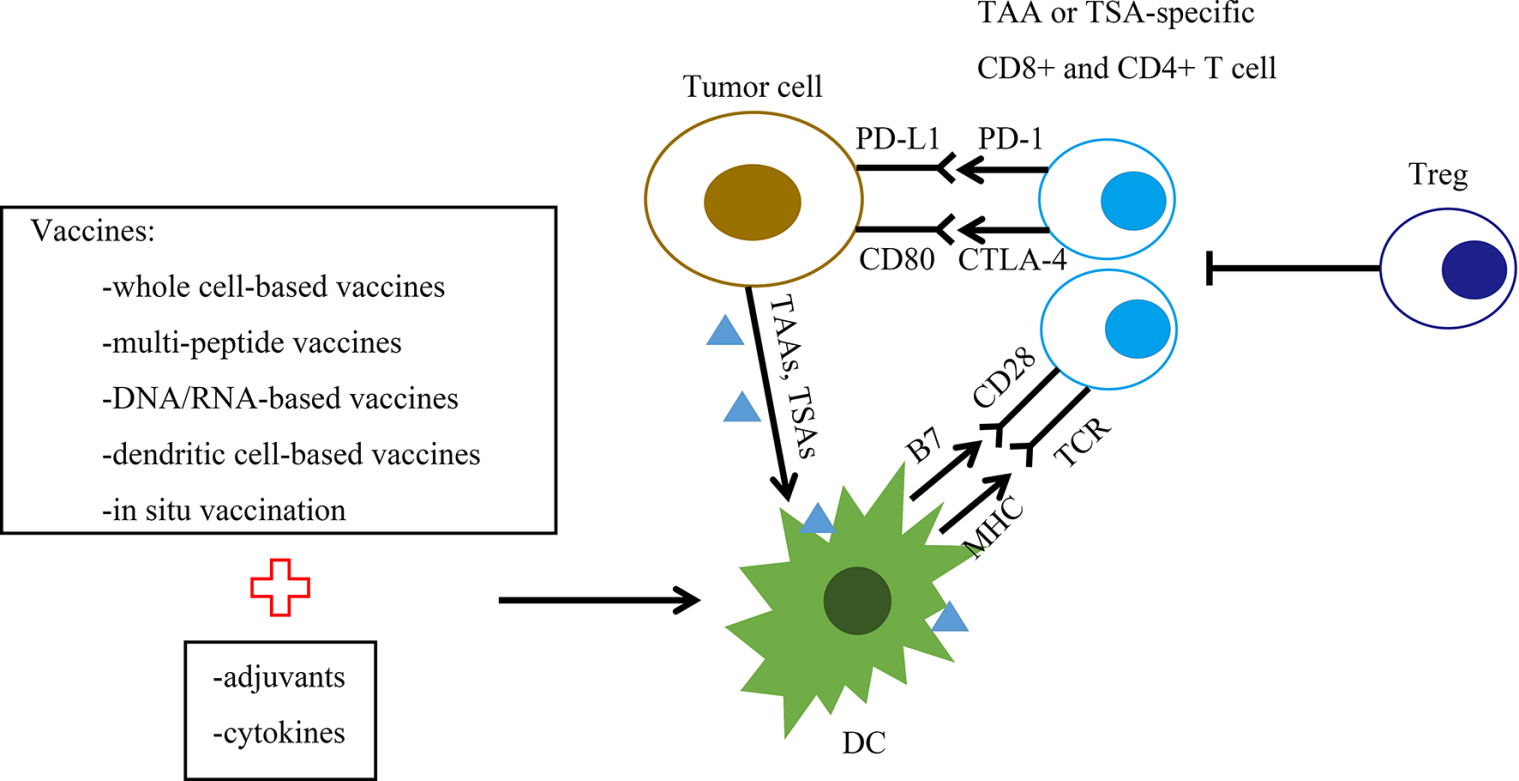
Graphic representation of the therapeutic breast cancer vaccine platforms and their mechanism of action. The figure summarizes the spectrum of vaccine targets and vaccine platforms for breast cancer. Vaccine platforms are whole cell-based vaccines, multipeptide vaccines, DNA/RNA-based vaccines, dendritic cell (DC)-based vaccines and *in situ* vaccination. DCs present processed tumor-associated antigen (TAA) or tumor-specific antigen (TSA) to CD4+ and CD8+ T cells. This interaction generates TAA/TSA-specific effector T cells, leading to the killing of tumor cells. B7, B7 protein; CD28, T cell costimulatory molecule CD28; CTLA-4, cytotoxic T lymphocyte-associated antigen 4; DC, dendritic cell; MHC, major histocompatibility complex; PD-1, programmed cell death protein 1; PDL-1, programmed death ligand 1; TAAs, tumor-associated antigens; TSAs, tumor-specific antigens; TCR, T cell receptor; Treg, regulatory T cell.

Therapeutic tumor antigens are divided into two main categories: tumor-associated antigens (TAAs) and tumor-specific antigens (TSAs) (9) (**Figure 1**). TAAs include tumor germline antigens, tumor differentiation antigens and overexpressed antigens (10). Tumor germline antigens, or cancer testis antigens, are expressed at high levels in the germinal cells of the testis, ovaries, and placenta and are not expressed in somatic cells under normal conditions (11). They are expressed in malignant cells of various cancer types, including BC. In BC, the expression of a number of cancer testis antigens has been reported, such as MAGE-A1 (12), NY-ESO1 (13) and KK-LC-1 (14). Serum antibodies against cancer testis antigens can be detected as useful biomarkers for predicting the clinical benefits of immunotherapy (14–16). Tumor differentiation antigens are proteins expressed in tumor cells and in normal tissue from which the tumor originates, such as Melan-A/Mart-1 (17), gp100 (18), PSA (19), CEA (20) and NY-BR-1 (21, 22). Overexpressed antigens are proteins expressed at low levels in normal cells and at high levels in cancer cells. The most common overexpressed antigens targeted in BC are HER2 (23), MUC-1 (24), hTERT (25) and survivin (26). TAA-based vaccines must be sufficiently immunogenic to activate the remaining low-affinity TAA-reactive T cells because central and peripheral immune tolerance mechanisms have removed T cells with strong TAA affinity.

TSAs are expressed specifically in tumor cells, mainly including oncoviral antigens and neoantigens (27). Neoantigens are products of genomic alterations and consist of simple point mutations that change single amino acids, frameshift insertion or deletion mutations, splice-site alterations that lead to exon skipping, structural alterations that lead to the formation of fusion proteins and other forms of collateral damage (28). Although there are thousands of genomic alterations in the process of tumor formation, only a handful of neoantigens succeed in eliciting antitumor immune responses. BC shows an intermediate genomic mutational load, and only a few cases benefiting from neoantigen-based treatment have been reported in BC (29). Since TNBC is recognized as a potential suitable subtype for immunotherapy, clinical trials of neoantigens are enrolling TNBC patients to evaluate the safety and induction of specific T cell responses. Clinical trials using autologous dendritic cells (DCs) pulsed with tumor-specific neoantigen (NCT04105582) or neoantigen DNA vaccine administered with durvalumab (NCT03199040) or personalized synthetic long peptide (SLP) neoantigen vaccine administered with durvalumab and nab-paclitaxel (NCT03606967) are currently enrolling TNBC patients. The neoantigen prediction process includes identifying tumor-specific somatic mutations and predicting major histocompatibility complex (MHC)-binding epitopes. Whole-exome sequencing is performed using tumor biopsy specimens and nonmalignant tissue samples to identify tumor-specific somatic mutations (30, 31). Tumor and germline DNA are compared to exclude germline mutations, while RNA sequencing provides additional information on mutated genes and confirms the mutation calls (32–34). Owing to human leukocyte antigen (HLA) restriction, various algorithm-based computational approaches have been developed to predict the binding of a tumor antigen to MHC molecules (35, 36). Peptides predicted with a moderate-to-strong HLA-binding affinity (IC50 < 150 nmol/l) are considered more likely to induce CD8+ T cell responses. Mass spectrometry-based immunopeptidomics can be used to identify neoantigens or to validate those predicted by in silico strategies. Recently, a new strategy based on using signaling and antigen-presenting bifunctional receptor (SABR) libraries was developed, enabling the identification of specific TCR-pMHC interactions (37).

In addition to TAAs and TSAs, multiple TME-targeting vaccine-based clinical trials are underway for patients with BC (38). Resident cells in the TME are likely more genomically stable than tumor cells. Pathological angiogenesis in the vascular TME can suppress effective immunotherapies. Multiple strategies targeting whole-cell endothelial cells (39), tumor blood vessel antigens (40), epidermal growth factor receptor (EGFR) (41), CD105 (42), platelet-derived growth factor receptor (PDGFR)-β (43) and vascular endothelial growth factor receptor (VEGFR) (44) have been tested in preclinical models of BC. A phase I study of pulsed DCs with tumor blood vessel antigens was completed recently (NCT02479230). Cancer-associated fibroblasts of the TME are vaccine targets as well. However, cancer-associated fibroblast vaccine strategies are all in the preclinical stage (45–47). Mads Hald Andersen et al. (48) designed an innovative investigational approach to target immune inhibitory pathways in the TME, modulating immune regulation. Therapeutic vaccination with long peptide epitopes is derived from proteins including indoleamine 2,3-dioxygenase (IDO), tryptophan 2,6-dioxygenase, arginase, and programmed death ligand 1 (PD-L1). Endogenous anti-regulatory T cells are activated because they recognize these peptides, and these pro-inflammatory cells are attracted to the TME, potentially altering tolerance to tumor antigens. Vaccinations against IDO or PD-L1 have been proven to be safe in clinical trials. Tryptophan 2,6-dioxygenase (TDO) is another enzyme involved in tryptophan degradation in the TME and is expressed in many cancers, including breast cancer, making it an interesting target for therapeutic vaccinations against the TME for BC. Vaccines are also currently being developed to target gene products associated with epithelial-mesenchymal transition (EMT) and cancer cells with stem-like characteristics (49, 50).

## Vaccine Delivery Platforms

Diverse vaccine platforms have now been evaluated in clinical trials, including whole cell-based vaccines, multipeptide vaccines, DNA/RNA-based vaccines, dendritic cell-based vaccines and *in situ* vaccination (**Table 1**).

**Table 1:** Comparison of different vaccine platforms.

Vaccine platforms	Mechanisms	Advantages	Disadvantages	Ref
Whole cell-based vaccines	Whole tumor cell lysates can be prepared by hypochlorous acid, ultraviolet B ray-irradiation, repeat cycles of freezing and thawing or hyperthermia	All tumor cells express a wide range of tumor-associated antigens Gene sequencing and bioinformatics predictive screening are not required Diminishes the chance of tumor escape	Complex and expensive production The immunogenicity is relatively poor	(51)
Multipeptide vaccines	Peptide vaccines contain tumor-specific epitopes that can be taken up and processed by antigen-presenting cells to activate T cell immune responses	Stable Safe Can be inoculated repeatedly Long peptides can stimulate both CD4+ and CD8+ T cell responses	The immunogenicity of synthetic peptide-based vaccines can be significantly affected by the delivery process	(52)
DNA/RNA-Based Vaccines	*In vitro* transcribed RNA or plasmid DNA encoding cancer antigens is introduced into the body, and cancer antigens are expressed by the host to induce antitumor response	Rapid and inexpensive production Mimics viral infection DNA vaccines have flexible platform for molecule engineering RNA vaccines have intrinsic adjuvant properties	RNA vaccine is susceptible to extracellular degradation by RNAses DNA vaccine has theoretical risk of host genome integration, relatively low immunogenecity	(53)
Dendritic cell-based vaccines	DC cells are stimulated with cytokines *in vitro* to become mature DCs upregulating costimulatory molecules, and mature DCs loaded with antigens migrate to lymph nodes resulting in the subsequent specific immune responses	Bypass conventional antigen presentation pathways	Time-consuming personalized process Less practical Hard to preserve	(54)
*In situ* vaccination	Manipulation of intratumoral myeloid cells by injecting immunomodulators and local ablative therapies which are used to release tumor antigens from the therapy-killed tumor cells such as radiation or combination with vaccines	Simple and cost-effective Minimal side effects Minimizes immune escape Adjuvant delivery is feasible and flexible	Requirement for intratumoral injection	(55)

### Whole Cell-Based Vaccines

Whole cell-based vaccines are derived from autologous or allogenic tumor cells (56). Immunizing BC patients with tumor cells isolated from the patient can circumvent the problems associated with antigen selection and epitope prediction. In addition, whole cell-based vaccines present the patient’s immune system with a wide variety of TAAs as immunogens. However, whole cell-based vaccines have shown relatively poor immunogenic potential (57). The immunogenicity can be increased by engineering tumor cell lines to secrete granulocyte-macrophage colony stimulating factor (GM-CSF), combined with strong adjuvants or cytokines (58, 59). In addition, whole cell-based vaccines in combination with chemotherapy may also exert synergistic antitumor effects. Autologous tumor cell vaccines (ATCVs) present a unique set of antigens, such as particular point mutations or fusion gene products, from a given patient’s own tumor (60–62). These antigens could help to launch a polyclonal response against a variety of tumor cells. However, the generation of ATCVs is patient specific with high complexity and high cost. Allogenic tumor cell vaccines, which typically contain two or three established human tumor cell lines, can be used as an alternative for the development of cell-based vaccines (56). In a phase I clinical trial enrolling 28 patients with stable metastatic breast cancer (mBC), the efficacy of a combination therapy using an allogenic GM-CSF-secreting BC vaccine along with chemotherapy was investigated (63). The vaccine was formulated from two HER2/neu+ mammary adenocarcinoma BC cell lines, SKBR3 and T47D. This vaccine was administered either alone or in sequence with common chemotherapeutic agents, such as cyclophosphamide and doxorubicin. The results suggest that the vaccine alone or in sequence with low-dose chemotherapy could induce an effective immune response. In another phase I study, a human leukocyte antigen (HLA)-A2+, HER2/neu(+) allogeneic MDA-MB-231 BC cell line was modified to express the costimulatory molecule B7-1 (CD80) and used as a vaccine to treat stage IV BC patients (64). Although this immunization strategy proved to induce tumor-specific immune responses in a minority of patients, no significant tumor regression was observed. In a single-arm feasibility study, an allogeneic HER2+ GM-CSF-secreting BC vaccine was given with low-dose cyclophosphamide and weekly trastuzumab in 20 patients with HER2+ mBC (65). This vaccination regimen was safe and demonstrated clinical effects in terms of objective response rate (ORR), progression-free survival (PFS), and overall survival (OS), with a trend toward longer PFS and OS in HER2-specific T-cell responders.

### Peptide Vaccines

The advantages of peptide vaccines include ease of synthesis and storage, safety, cost-effectiveness, and tolerable side effects. The great limitation for peptide-based vaccines is the possibility of insufficient immunogenicity, which makes a great need for a suitable adjuvant to produce an efficient response. The expression of antigen epitopes within the tumor bed can be heterogeneous, while the immune response may be limited to a few epitopes. Multipeptide vaccines formulated from MHC class I-restricted TAAs are being tested for their antigen-specific immune response in clinical trials (66–70). Peptides with epitopes can bind directly to MHC class I molecules on the surface of antigen-presenting cells without cross-presentation, but they often result in only low-level, short-lived responses without the help of CD4+ T cells. CD4+ T cells can enhance the tumoricidal activity of other antitumor effector cells, such as CD8+ T cells and macrophages. Some CD4+ subsets influence angiogenesis to facilitate the infiltration of CD8+ T cells, in addition to direct cytotoxic functions (71). There are attempts to activate both CD4+ and CD8+ T cells by using multivalent synthetic long peptides (SLPs) containing both MHC class I and class II epitopes (72). SLP vaccines offer several advantages. They are not able to bind directly to MHC class I so that they have to be processed by DCs (73). SLP vaccines increase the duration of *in vivo* epitope presentation in the antigen-draining lymph node (74), which is shown to be important for clonal expansion (75) and for interferon-γ production by CD8+ T cells (76), and harbor both CD4+ and CD8+ T cell epitopes, ensuring a balanced CD4/CD8 response. Some well-designed peptide vaccines will be discussed in the 4th part of this review. In addition, delivery systems have been applied to improve antitumor immunity. Among them, nanomaterials, such as liposomes, micelles, dendrimers, microneedles, proteins, polymer-based conjugates, the B-subunit of Shiga toxin (STxB), and polyactin A (PAA), are under investigation to convey and release antigens and immunostimulatory molecules (77).

### DNA/RNA-Based Vaccines

DNA or RNA-based vaccines are easy to design and can encode multiple epitopes. DNA vaccines have good stability and can be rapidly and easily modified. Plasmid DNA vaccines can be integrated with additional immune modulators to elicit a maximal immune response (78). Most DNA-based cancer vaccine studies have targeted TAAs, such as HER2/neu and mammaglobin-A (MAM-A), in BC. The first clinical trial of a HER2/neu DNA vaccine evaluated the efficacy and tolerability of the vaccine in humans. The HER2/neu DNA vaccine was administered with low doses of interleukin-2 (IL-2) and GM-CSF in mBC patients in a pilot clinical trial, even though no significant T cell response was elicited (79). Currently, two phase I clinical trials of HER2/neu DNA vaccines are active (NCT00393783 and NCT00436254). The MAM-A DNA vaccine was also investigated in mBC in a phase I clinical trial. This vaccine was safe and succeeded in eliciting MAM-A-specific CD8+ T cell responses. PFS was improved in vaccinated patients, although the sample size was low (n=14) (80). Additionally, a clinical trial using a neoantigen DNA vaccine to treat TNBC was launched (NCT03199040). RNA vaccines are designed to enter the cytosol and thus avoid safety concerns related to integration into the host cell genome. RNA-based vaccines have an inherent function through Toll-like receptor 3 (TLR3), TLR7 and TLR8 stimulation to provide an adjuvant effect. However, RNA is very unstable, so delivery systems such as nanoparticles and liposomes are challenging. Viral vectors can be used to deliver nucleic acid vaccines to enter the cytosol. However, the production of antibodies against viral vectors attenuates the efficiency. PANVAC (containing transgenes for CEA, MUC-1 and 3 T cell costimulatory molecules) is a well-studied poxviral vaccine. For the 12 mBC patients, 5 patients had stable disease (SD) by RESIST lasting ≥ 4 months, with one patient having a complete response (CR) and remaining on study for ≥ 37 months (81).

### Dendritic Cell-Based Vaccines

DCs are professional antigen-presenting cells that can process exogenous and endogenous antigens and present them to stimulate naïve T lymphocytes through the MHC I and II pathways. Therefore, DCs play a crucial role in the initiation of the primary response and induction of the antitumor-specific immune response. Most cancer vaccines are greatly dependent on the activation of DCs. Peptide-pulsed DCs have superiority in inducing antitumor responses compared to peptide vaccines with adjuvants (82). In a pilot study, autologous DCs were pulsed with HER2/neu- or MUC1-derived peptides to generate a DC-based vaccine. Ten patients suffering from advanced BC and ovarian cancer showed a strong immunogenic response with no side effects (83). A HER2 intracellular domain (ICD) protein-containing DC vaccine was tested in disease-free BC patients. Six patients out of seven had circulating anti-HER2 ICD antibodies, and all patients were alive and disease free at 4.6-6.7 years of follow-up (84). Autologous DCs were also pulsed with patient-derived tumor cells or cell lysates to facilitate a strong immunogenic response (85–87). However, ex vivo generation of DCs is complicated, and it is costly and time-consuming to generate the large number of DCs required for vaccination. The demanding production process of DC vaccines and lack of improvement in clinical benefits limit their application in the clinic.

### *In Situ* Vaccination

*In situ* vaccination (ISV) refers to inducing and stimulating an immune response specially at the tumor site (88). ISV uses the tumor itself as the antigen source and should be defined as a treatment process or strategy. There are several advantages of ISV. It is simple and cost-effective with minimal side effects, and it utilizes all tumor antigens in the tumor which minimizes immune escape. There is no need to identify antigens and adjuvant delivery is feasible and flexible. Besides, there is a great chance to obtain synergistic effect with other therapies (55). One limitation may be due to intratumoral injection, because some internal tumors will not be accessible to injection. As to breast cancer, the primary tumor is superficial, skin and regional lymph node recurrence is common. Therefore, breast cancer is quite accessible to injection, making it a good candidate for ISV.

Food and Drug Administraion (FDA) has approved a number of ISV-based cancer immunotherapies, such as Bacilus Calmette-Guerin (BCG) for *in situ* vaccination, toll-like receptor agonists for *in situ* vaccination, oncolytic virus for *in situ* vaccination, and *in situ* vaccination with cytokines and immune checkpoint blockade. ISV involves manipulation of intratumoral immune cells by injecting immunomodulators (89) and local ablative therapies which are used to release tumor antigens from the therapy-killed tumor cells (90). Besides, local treatment with vaccines and adjuvant is another option to provoke immune system in situ (91). The combination of ISV with other immnutherapies is likely to provide the optimal local control and systemic antitumor effect. Yokoi et al. treated mammary tumors with *in situ* immunomodulation consisting of intratumoral injections of Fms-like tyrosine kinase 3 receptor ligand to mobilize conventional type-1 dendritic cells (cDC1s), local irradiation to induce immunogenic tumor cell death, and TLR3/CD40 stimulation to activate cDC1s. Circulating effector T cells and CD8+ T cells infiltrated into metastatic brain lesions were increased and resistance to anti-PD-1 therapy was overcome, resulting in improved survival. Radiation can elicit systemic response which is known as abscopal effect, and the potential mechanism is to release tumor antigens in the process of ISV (92). Numerous clinical data supported the concept of radiation as an important part during *in situ* vaccine treatment (93–95), and clinical trials are underway investigating combination therapy of radiation with other immunotherapies (91). Combination therapy with noninvasive low intensity focused ultrasound and ablative radiation therapy was reported to generate an *in situ* tumor vaccine as well (96). Like radiation, heat (hyperthermia) has been used to damage targeted tumors and could be further combined with ISV (97). More approaches will be integrated into future multi-modality therapy.

### Therapeutic Vaccines for Breast Cancer in Clinical Trials

The treatment for BC at different stages includes neoadjuvant therapy, adjuvant therapy for early BC, rescue therapy and maintenance therapy for advanced BC. Therapeutic vaccines for BC at different stages are summarized.

### Neoadjuvant Setting

Disease at an early stage presents with a more intact immune system and a lower tumor burden, possibly affording vaccines the potential to confer a more favorable outcome. Therapeutic vaccines in the neoadjuvant setting are the theoretically most likely method to optimize the immune microenvironment and improve prognosis. Cancer treatment starts with modulation of the microenvironment and promotion of antitumor immunity before any inhibition occurs to the immune system.

Mucin or MUC-1 is a transmembrane glycoprotein that is expressed in the lung, colon, breast, ovary, pancreas and other cancer tumor cells. MUC-1 is considered a promising candidate for vaccine development in BC. Tecemotide is a synthetic 27 amino acid lipopeptide used as an MUC-1 immunogen that is applied in clinical trials of prostate, NSCLC and colon cancer with promising effects. In a prospective, multicenter, randomized 2-arm academic phase II trial (ABCSG 34), tecemotide was added to neoadjuvant standard-of-care treatment in early BC patients. Approximately 400 patients with early BC were recruited into this trial. No significant difference was observed in residual cancer burden or overall pathological complete response (pCR) rates between the two groups. This trial demonstrated that MUC-1-based vaccination strategies are safe but did not show an improved treatment effect when added to standard treatment in the neoadjuvant setting (98). However, disease-free survival data are still premature and may provide further information. Interestingly, tumors which achieved a residual cancer burden (RCB) 0/I and a pCR had a higher concentration of intratumoural and stromal tumor-infiltrating lymphocytes in the pre-therapeutic biopsy than those which did not. Several ongoing studies address vaccines for BC in the neoadjuvant setting (NCT03387553, NCT02204098, NCT03564782, NCT03572361, NCT04144023). Further data are needed to determine whether neoadjuvant vaccine therapy can reduce the risk of recurrence and prolong relapse-free survival.

### Adjuvant Setting

Further immune elimination of subclinical lesions is an important function of vaccines for BC after tumor resection. There have been a number of clinical studies of preventive vaccines in the field of adjuvant therapy.

In a pilot clinical trial of oxidized mannan–MUC-1 (M-FP) for the treatment of patients with stage II BC, the follow-up at 12-15 years showed that the recurrence rate was 12.5% (2/16) in the vaccine group compared with 60% (9/15) in the placebo group. M-FP also benefits the overall survival of stage II BC patients (99). In a phase II clinical trial (NCT02764333), a folate receptor alpha-based vaccine (TPIV200) was investigated in TNBC patients. In this trial, an immunologic response was elicited, and more data has not been exposed.

Peptide vaccines for HER2 have been explored in the adjuvant setting. The E75 peptide vaccine (nelipepimut-S), an HLA-A2/A3-restricted extracellular HER2-domain-derived peptide, is an MHC class I epitope (100). A series of trials in the adjuvant setting were conducted at approximately E75, demonstrating not only a good safety profile of the E75 peptide vaccine but also a superiority of immune response in BC patients with low HER2 expression than vaccinated patients with high levels of HER2 expression (101). Mittendorf et al. further examined schedule optimization according to lymph node (LN) status and risk of disease recurrence in a phase I/II clinical trial (69). Analysis of disease-free survival (DFS) revealed that patients who had tumors with low HER2 expression (immunohistochemistry score 1+ or 2+ with fluorescence *in situ* hybridization negativity) and had positive lymph nodes benefited the most from vaccination therapy. In a phase I/II trial, 187 LN-positive and high-risk LN-negative breast cancer (IHC score 1-3) patients were evaluated in the adjuvant setting. E75 patients with GM-CSF versus placebo were administered to 108 patients with HLA-A2/3- and 79 HLA-A2/3-negative patients, respectively. The results concluded that the 5-year DFS was improved for those who received E75 with respect to controls (89.7% vs 80.2%, P=0.08) (102). Given these promising data, in phase III clinical trials, the study assessed the effects of vaccination with E-75 plus subcutaneous GM-CSF relative to placebo in LN+ BC patients with low expression of HER2 in the adjuvant setting (103). However, no significant difference was found in DFS between the vaccine group and the control group, leading to the termination of the trial. Future clinical trials should be carried out to study the combination of vaccines with other medications. Several studies were conducted combining traustuzumab plus E75 in hope of a synergistic effect of active immunotherapy and passive immunotherapy (104). In phase IIb, multicenter, randomized, single-blinded, controlled trial (NCT01570036), the efficacy of the combination with E-75 plus traustuzumab was evaluated in patients with HER2 low-expressing BC in the adjuvant setting. No significant difference in DFS was seen in the HER2 low-expressing BC; however, significant clinical benefit was seen in patients with TNBC (105). These findings warrant further investigation in a phase III randomized trial. GP2 is a 9 amino acid-long peptide vaccine derived from the transmembrane domain of the HER/neu protein. It binds to the HLA-A2 molecule but has poor binding affinity compared to E75 (106). A phase II clinical trial was conducted to investigate GP2 vaccine efficacy in preventing recurrence in LN+ and high-risk LN- HER2 breast cancer patients (IHC 1+–3+) in the adjuvant setting. The results of the primary analysis did not show a significant difference in response to the vaccine compared to the control group in the rate of recurrence (70). However, patients who were vaccinated with GP2+GM-CSF had a significant increase in their delayed type hypersensitivity (DTH) reaction compared to pre-vaccination (p<0.001), the post-vaccination response was significantly greater in vaccinated patients than in control patients (p<0.001). In addition, ex vivo immune responses were assessed by phenotypic clonal expansion assays and by T cell functional assays. The GP2+GM-CSF vaccine induced significant increase in both clonal expansion as well as improved CTL function compared to pre-vaccine levels while GM-CSF alone had no such effect. A post for a prospective, randomized, single-blinded, placebo-controlled, multicenter phase IIb clinical trial was presented during the 2020 San Antonio Breast Cancer Symposium (SABCS) on December 09, 2020. This trial was completed in 2018, and Kaplan–Meier analysis of DFS for patients treated with GP2 immunotherapy showed 100% survival (0% breast cancer recurrence, p=0.0338) in the HER2/neu-positive adjuvant setting after a median of 5 years of follow-up. Greenwich LifeSciences announced an update of the GP2 phase III clinical trial design at the 2021 American Association for Cancer Research (AACR) annual meeting.

### Metastatic Setting

Most mBC cannot be cured by surgery and is highly dependent on systemic therapy. Therapeutic vaccines can be used in combination with other therapies as part of rescue therapy, and other studies are exploring their value as maintenance therapy for advanced breast cancer.

Therapeutic vaccines for rescue therapy for mBC have rarely been reported. Wilms tumor 1 (WT-1) is a protein with transcription factor activity involved in the maintenance of tissue homeostasis, possibly as an oncogene in BC. In a phase I clinical trial, WT-I vaccination activated WT-1-specific cytotoxic T lymphocytes (CTLs) and resulted in cancer regression with a good safety profile in 2 patients with BC with overexpression of the WT-1 gene and HLA-A*2402-possibility (107). Yang et al (108) enrolled 10 patients with advanced cancers, including mBC, and treated them with a DC-based WT-1 vaccination. Two patients had a partial response (PR), and three patients had stable disease (SD) with a disease control rate up to 50%. WT-1-specific CTL responses were enhanced in patients. CEA is overexpressed in BC and has attracted much attention as a target of vaccines. In a pilot study, the recombinant PANVAC poxviral vaccine (containing transgenes for CEA and MUC-1 and three T cell costimulatory molecules) was tested in 12 heavily pretreated metastatic BC patients. One patient demonstrated a CR lasting >37 months, and 4 patients had SD lasting >4 months. The median time to progression (TTP) was 2.5 months, and the median OS was 13.7 months (81). In another open-label phase II clinical trial, 48 patients with mBC were enrolled to receive treatment with either docetaxel with PANVAC or docetaxel alone. The median PFS was 7.9 months in the vaccination group vs 3.9 months in the docetaxel alone group, but the difference was not significant (p=0.09) (109). There was also no statistical correlation seen between the generation of TAA-specific immune responses in peripheral blood mononuclear cells and time to progression in either group. Takahashi et al. (110) developed a novel regimen of personalized peptide vaccination (PPV), in which vaccine antigens were selected and administered from a pool of 31 different peptide candidates based on the pre-existing immunoglobulin G (IgG) responses specific to peptides before vaccination. Based on previous results in cancer patients, they conducted a phase II study of PPV for metastatic recurrent breast cancer patients who had failed standard chemotherapies. Boosting of CTL and/or IgG responses was observed in most of the patients after vaccination. In addition, three CR cases and six PR cases were observed, irrespective of the BC subtypes. In a more recent early phase II study including 14 advanced metastatic triple-negative breast cancer (mTNBC) patients, the treatment protocol consisted of a weekly vaccination of mixed 19-peptide cancer vaccine monotherapy for 6 weeks. An increase in peptide-specific IgG was observed in all patients. The median OS was 11.5 months in all 14 patients and 24.4 months in the patients who completed the vaccination (111). Human telomerase reverse transcriptase (hTERT) is nearly universally overexpressed in human cancers and contributes critically to oncogenesis. A phase I clinical trial was performed to evaluate the HLA-A2-restricted hTERT I540 peptide presented with keyhole limpet hemocyanin (KLH) by ex vivo-generated autologous DCs. hTERT-specific T lymphocytes were induced in 4/7 patients after vaccination. PR was seen in 1 patient in association with the induction of CD8+ tumor infiltrating lymphocytes (112). In conclusion, although no prospective large-sample studies have confirmed the efficacy of therapeutic vaccines in the rescue therapy of advanced BC, some studies have preliminary results suggesting their effectiveness and possible prospects.

Immunosurveillance using therapeutic vaccines to trigger active immunity when remission is achieved through rescue therapy such as radiotherapy or chemotherapy suggests novel opportunities for both therapeutic and prophylactic vaccine strategies for cancer treatment. MAM-A is overexpressed in 40-80% of breast tumors. Tiriveedhi et al. (80) enrolled 14 mBC patients with stable disease and treated them randomly with the MAM-A vaccine or placebo in a phase I clinical trial. Although this trial was not powered to evaluate PFS, improved PFS was seen in vaccinated patients. A significant increase in the frequency of MAM-A-specific CD8+ T cells (0.9% + 0.5% vs 3.8% + 1.2%; p<0.001) and an increase in the number of MAM-A-specific IFNγ-secreting T cells (41 + 32 vs 215 + 67 spots per million cells (spm); p<0.001) were observed. Increased Siayl-TN (STn) expression, which is a carbohydrate epitope found on a variety of glycoproteins, including MUC-1, has been proven to be associated with the progression and poor prognosis of BC (113). Miled et al. (114) conducted the largest phase III clinical trial in 1028 mBC patients across 126 centers. Patients were administered a vaccine made of STn conjugated to the carrier protein KLH versus placebo. Although clinically significant antibody titers specific for STn were produced in patients, no significant improvement in TTP or OS was observed (115). Ibrahim et al. conducted a subgroup analysis in which patients who were also on endocrine therapy (ET) had longer TTP and OS than the control group. Moreover, vaccinated patients on ET with higher antibody responses had longer OS (41.3 vs 25.4 months; p=0.0147). In an open-label prospective study, 19 patients with mBC refractory to at least one conventional therapy were treated with the hTERT peptide vaccine, and hTERT-specific CD8+ T cells were detected after vaccination in the peripheral blood of patients and exhibited effector functions *in vitro*, including proliferation, IFN-gamma production, and tumor lysis. In this small sample study, the median OS was significantly longer in patients who achieved an immune response to hTERT peptide than in patients who did not (116). All the results above suggest that therapeutic vaccines are a potentially feasible option for maintenance therapy of advanced BC, but no mature vaccine has been proven to be beneficial in a large-sample clinical trial. Another important issue that should be considered is that the essential immune capability to recognize and activate antigens should be conserved before vaccination.

## Future Perspectives

### Tumor Stage Specific Vaccine Strategy

During cancer clonal evolution, both selection and neutral growth may progress simultaneously within the same tumor, but both styles of tumor progression may alter dynamically over time (117). Metastatic BC shows an increase in mutational burden and clonal diversity compared to early BC because genomic alterations are acquired during the evolution of cancers from their early stages (118). A multitude of epigenetic mechanisms, including DNA methylation, chromatin remodeling and posttranslational modification of histones, contribute to diversity within tumors, and the heterogeneity becomes extensive. Intratumor heterogeneity (ITH) is a key factor contributing to the lethal outcome of cancer, therapeutic failure, and drug resistance. Some claim that tumors with high heterogeneity may generate neoantigens that attract immune cells (119), but others argue that immune cells provide selection pressure that shapes tumor heterogeneity. High heterogeneity tumors are associated with higher subclonal diversity, less immune cell infiltration, less activation of the immune response, and worse survival in BC (120). Immune-infiltrated tumor regions exhibit either HLA loss of heterozygosity (LOH) or depletion of expressed neoantigens, which will eventually make it increasingly difficult to treat mBC with an immune strategy (120–122). Finding the right target antigen and intervening at the right time are the most important issues of active immunotherapy. The continuous evolution of the immune microenvironment during tumorigenesis also suggests that different modes of treatment should be considered at different stages.

Neoantigen profiles keep changing while tumor-specific mutations change during tumorigenesis and progression. Therefore, individual immune status, clonal heterogeneity and stage of disease should be fully considered, and time specificity should be realized.

### Universal Vaccines

Optimal antigens should be developed from publicly mutated genes or high frequency overexpressed genes that are shared by a number of patients. A punch of such public antigens that are consumed to cover most patients with one type of cancer can be used to develop public vaccines, also named universal vaccines (123). Universal vaccines have the great advantage of convenient production and reduction in cost. In addition, preprepared vaccines that can be quickly inoculated into patients also save time and are more practical. The efficacy of universal vaccines should be ensured. One important problem should be considered except for the restriction of MHC molecules. That is, the proportion of tumor antigen expression in the population. Although more than 900,000 neoantigens have been identified through a wide examination of 20 tumor histotypes, only 24 neoantigens among a tiny fraction of patients have the potential to become public vaccines (124). Therefore, it is more feasible to develop public vaccines based on TAAs. Public vaccines have broad coverage and can improve the immune surveillance function of individuals to prevent tumor metastasis and recurrence. It is theoretically more suitable for the stage of neoadjuvant and adjuvant therapy.

From a single genome point of view, improving the antitumor effect of tumor-specific T cells and memory T cells is important for designing therapeutic vaccines. Personalized therapeutic vaccines targeting trunk or driver mutations are more effective and have a more comprehensive antitumor effect than those targeting companion or passenger mutations. In addition, the option of designing vaccines needs to be weighed between selecting a large number of target antigens to avoid immune escape and selecting antigens with good immunogenic potential.

### Concurrent Therapies With Vaccination

Conventional therapies such as chemotherapy, radiotherapy and targeted therapy constantly promote the emergence of new subclones of tumor cells as a result of the pressure of clonal evolution, resulting in treatment failure. Immunotherapy, as a new therapeutic strategy, has a totally different effect on tumor heterogeneity from conventional therapy. However, patients who have received multiline conventional therapies can hardly benefit from immunotherapy. How to maximize the therapeutic effect of immunotherapy by rational arrangement of comprehensive therapy is an important direction in the future. In addition, how to exert antitumor effects of therapeutic vaccines synergistically with various therapeutic means is a hotspot. It was reported that sequential treatment with vaccine and PD-1 blockade was more effective than a simultaneous treatment regimen (125). In the PACIFIC trial, when durvalumab therapy was initiated within 14 days of completing chemoradiotherapy, better progression free survival was observed than when it was initiated after 14 days (126). Thus, timing is an important factor in obtaining abscopal effect and the optimal scheduling of vaccines, immunotherapy, radiation and chemotherapy needs to be clearly established, ideally through clinical trials. The TME is a major reason for the disappointing clinical results in addition to tumor-intrinsic resistance mechanisms, so an inflammatory TME is needed for sterile immunity (38). Except for what we have mentioned above about TME-targeting vaccines, *in situ* TME modulation strategies include stimulation of professional antigen presenting cells, combination with checkpoint inhibitors and depletion of regulatory T cells (Treg cells). PVX‐410 (PVX) is a multipeptide vaccine targeting X‐Box Binding Protein 1 (XBP1), and CD138 is overexpressed in TNBC. The synergistic effects of PVX‐410 and ICI pembrolizumab will be evaluated in a clinical trial (NCT03362060) for TNBC. Another phase I clinical trial (NCT02826434) tested the synergistic effects of durvalumab and PVX‐410 for TNBC. In this trial, the levels of CD8+ CTLs increased in patients 14 weeks after the first injection. The combination therapy strategy to work together with vaccines will include, but is not limited to, ICIs, antiangiogenic therapy, epigenetic regulation therapy, low intensity focused ultrasound (55) and conventional chemoradiotherapy. Cyclophosphamide to block Treg cells has been evaluated as a vaccine adjuvant in clinical trials (NCT03012100, NCT02938442). Several other ongoing trials are further assessing the application of various promising vaccination therapies in early and metastatic disease (**Table 2**).

**Table 2:** Ongoing trials of tumor vaccine-based combination therapy for BCs (data from ClinicalTrials.gov).

Drug Regimen	NCT.gov Identifier	Sample Size	Phase; Status	Population
Neoantigen DNA Vaccine Durvalumab	NCT03199040	10	I; ANR	Clinical Stage T1c-T4c, Any N, M0 TNBC Prior to Neoadjuvant Chemotherapy, with Residual Invasive BC after Neoadjuvant Therapy
VRP-HER2 Pembrolizumab	NCT03632941	39	II; R	Advanced HER2-overexpressing BC
PVX-410 Pembrolizumab	NCT03362060	20	I; ANR	HLA-A2 + Metastatic TNBC
Galinpepimut-S Pembrolizumab	NCT03761914	90	I/II; R	Advanced Tumors including Advanced TNBC
RO7198457 Atezolizumab	NCT03289962	770	I; R	Advanced Tumors including Advanced TNBC
PVX-410 Durvalumab Hiltonol	NCT02826434	22	Ib; ANR	HLA-A2 + Subjects Following Standard Treatment of Stage II or III TNBC
Multiepitope Folate Receptor Alpha Peptide Vaccine Cyclophosphamide	NCT03012100	280	II; R	Stage I-III TNBC
NeuVax Vaccine Trastuzumab	NCT02297698	100	II; ANR	Stage I-III Noninflammatory, HER2+ High-risk BC
A Peptide Mimotope-based Vaccine P10s-PADRE with MONTANIDE™ ISA 51 VG Doxorubicin Cyclophosphamide Paclitaxel	NCT02938442	102	I/II; R	Stage I, II or III TNBC
AE37 Peptide vaccine Pembrolizumab	NCT04024800	29	II; ANR	Advanced TNBC
Dendritic Cell Vaccine Neoadjuvant Chemotherapy	NCT03387553	30	I; R	HER-2/neu Positive Invasive BC during Neoadjuvant Therapy
Anti-HER2/HER3 Dendritic Cell Vaccine Recombinant Interferon Alfa-2b Celecoxib Pembrolizumab	NCT04348747	23	IIa; NYR	Patients With Asymptomatic Brain Metastasis From TNBC or HER2+ BC
Personalized Synthetic Long Peptide Vaccine Carboplatin Durvalumab Gemcitabine Hydrochloride Nab-paclitaxel Tremelimumab	NCT03606967	70	II; R	Advanced TNBC
Multiepitope HER2 Peptide Vaccine TPIV100 Pertuzumab Trastuzumab	NCT04197687	480	II; R	HER2 Positive, Stage II-III BC in Patients With Residual Disease After Chemotherapy and Surgery
pUMVC3-IGFBP2-HER2-IGF1R Plasmid DNA Vaccine Paclitaxel Trastuzumab Pertuzumab	NCT04329065	16	II; R	BC during Neoadjuvant Therapy
Brachyury-TRICOM Entinostat M7824 Ado-trastuzumab emtansine	NCT04296942	65	I; R	Advacned BC
*In Situ* Vaccination With Flt3 L, Radiation, and Poly-ICLC Pembrolizumab	NCT03789097	56	I/II; R	Advanced, Measurable, Biopsy-accessible Cancers including BC
*In Situ* Vaccination Durvalumab Tremelimumab	NCT02643303	58	I/II; ANR	Advanced, Measurable, Biopsy-accessible Cancers including BC

ANR, active; not recruiting; NYR, not yet recruiting; R, recruiting.

## Conclusion

In recent years, the application of therapeutic vaccines has been gradually accepted in the field of BC, but both the candidates and the efficacy need further study. Increasing attention has been given to the use of therapeutic vaccines to modulate the immune microenvironment and fully mobilize the body’s own immune system for active immunotherapy. However, the exploration of therapeutic vaccines for BC is still in the early stage and is bound to be long based on considering the stage of disease, personal immune status and clonal heterogeneity. Fully combining therapeutic vaccines with not only ICIs but also other multiple treatment methods may take great advantage in the future treatment of BC.

## Author Contributions

The corresponding authors are responsible for ensuring that the descriptions are accurate and agreed by all authors. Authors have contributed in multiple roles. LZ is responsible for writing original draft and literature search. XZ is responsible for literature search. HS is responsible for literature search. LX is responsible for conceptualization and supervision. BL is responsible for review and editing for original draft and supervision. All authors contributed to the article and approved the submitted version.

## Funding

This work was funded by a grant from the National Natural Science Foundation of China (Grant No. 81803093). The funding sources had no role in the study design, data collection, data analysis, data interpretation, or writing of the report.

## Conflict of Interest

The authors declare that the research was conducted in the absence of any commercial or financial relationships that could be construed as a potential conflict of interest.

## Publisher’s Note

All claims expressed in this article are solely those of the authors and do not necessarily represent those of their affiliated organizations, or those of the publisher, the editors and the reviewers. Any product that may be evaluated in this article, or claim that may be made by its manufacturer, is not guaranteed or endorsed by the publisher.
